# Training Programs Designed for Muscle Hypertrophy in Bodybuilders: A Narrative Review

**DOI:** 10.3390/sports8110149

**Published:** 2020-11-18

**Authors:** Ragami C. Alves, Jonato Prestes, Alysson Enes, Wilson M. A. de Moraes, Thiago B. Trindade, Belmiro F. de Salles, Alan A. Aragon, Tacito P. Souza-Junior

**Affiliations:** 1 Metabolism, Nutrition and Resistance Training Research Group, Federal University of Paraná, Curitiba 81531-980, Brazil; ragami1@hotmail.com (R.C.A.); tacitojr@ufpr.br (T.P.S.-J.); 2 Department of Physical Education, Catholic University of Brasilia, Brasilia 71966-700, Brazil; jonatop@gmail.com (J.P.); wmaxnutri@gmail.com (W.M.A.d.M.); ttrindade@me.com (T.B.T.); 3 School of Physical Education and Sports, Federal University of Rio de Janeiro, Rio de Janeiro 21941-599, Brazil; b.desalles@stlab.com.br; 4 Department of Family & Consumer Sciences, California State University, Northridge, CA 91324, USA; alaneats@gmail.com

**Keywords:** bodybuilding, resistance training, skeletal muscle hypertrophy, off-season, pre-contest

## Abstract

Bodybuilding is a sport that requires adequate training strategies in order to maximize skeletal muscle hypertrophy. The purpose of the present review was to perform a narrative assessment of the training routines designed for muscle hypertrophy used by bodybuilders. A search was carried out in the databases Pubmed/MEDLINE, Scielo, EBSCO, LILACS, SportDiscus, Web of Science, and CINAHL with the words “Resistance training” and “hypertrophy” in bodybuilders and their variations that involve the respective outcomes. Fourteen studies were identified that investigated the long-term training routines of bodybuilders. These studies demonstrate a pattern in the training organization, whereby there is a separation of training into four distinct periods: off-season, pre-contest, peak week, and post-contest. Each period has a specific spectrum of intensity load, total training volume, and exercise type (multi- or single-joint). We conclude that bodybuilding competitors employed a higher intensity load, lower number of repetitions, and longer rest intervals in the off-season than pre-contest.

## 1. Introduction

Bodybuilding is a sport that involves performing a series of poses on stage where judges rank each competitor on aesthetic appearance based on muscular mass, symmetry, and definition [1,2]. The athletes that present superior scores in a greater number of poses prevail as the winner [1]. Based on these characteristics, the training practices of bodybuilders prioritize strategies intended to maximize skeletal muscle hypertrophy. Moreover, the degree of muscle hypertrophy and definition are category-dependent—for example, males participating in classic bodybuilding present less muscle volume and extreme muscle definition, while senior bodybuilders present higher muscle volume accompanied by muscle definition [3]. These categories and others, such as Men’s Physique and Classic Physique, are mainly differentiated by the amount of muscle mass, since on the stage it is expected that all athletes show as low a body fat as possible. Each category has proposed and specific rules dictating bodybuilders’ goals; in categories such as Classic Bodybuilding, the athlete’s height determines the body mass that must be shown on contest day. Classic Bodybuilding athletes have some difficulties in reaching a high muscle volume, due to the established rules for this category. Hence, muscle definition may be the most important criterion for this category. In this sense, we can speculate that these differences among bodybuilding categories may influence how athletes develop their training routines.

The American College of Sports Medicine [4] establishes guidelines for “healthy adults”, considering the training status and goals of the practitioner. With these guideline, it has been widely reported that a training intensity between 60% to 70% for one repetition maximum (1 RM) with multiple sets (3–6 sets) in a zone of 6–12 repetitions with rest intervals of 1–3 min between sets promotes increases in the muscle cross-sectional area (CSA) [4]. Although the specific resistance training (RT) protocols are for different purposes, the current guidelines fail to contemplate a range of training strategies commonly used by bodybuilders. This guideline is generalist, considering a “healthy adults” population and not bodybuilders, who have nutrition and recovery maximized to be ready for the next training session. In addition, considering some aspects in bodybuilders’ routines, such as nutrition and the use of pharmacological ergogenic aids, specific training routine aspects for maximum strength gains and muscle hypertrophy development remain undetermined [5,6].

Furthermore, RT designed for bodybuilders and trained subjects should be systematically organized to avoid plateau and maintain stimulus effectively, ensuring a progressive overload with the adequate managing of training loads (i.e., periodization), helping to manage the imposed stress of training sessions and avoiding non-functional overreaching or overtraining development in the long term [7,8]. However, we know that many bodybuilders do not necessarily train according to these recommendations [2]. This can be as ascertained through testimonials and interviews from professional athletes or their coaches. These reports also highlight the wide variability of strategies utilized in training to increase muscle mass.

Bodybuilders self-report a separation of training into two distinct phases: off-season (OFF) and pre-season (PRE) [1]; it has been argued that a peak week and a post-competition period may have distinctive strategies for the large OFF and PRE. In the OFF phase, these athletes routinely use split training routines, with muscle groups typically trained once or twice a week [9]. Generally, the PRE phase is initiated 20–12 weeks before the competition, whereby the main focus is on reducing body fat to extremely low levels [10]. During this period, many bodybuilders report a high volume of aerobic exercise, accompanied by sessions of RT with reduced loads [9]. Others maintain high loads and choose to increase the volume of the RT session while performing little aerobic exercise [1]. Therefore, the structures of training programs among bodybuilders are distinct, as not all follow the same periodization schemes and associated manipulation of RT variables.

The lack of direct, objective research on bodybuilding training practices is reinforced by a narrative review by Helms et al. [11], who endeavored to provide evidence-based guidelines for training practices in natural bodybuilders. However, the discussion was based on research findings in which the participants were not bodybuilders. In the cited and discussed studies involving bodybuilders, the subjects did not report their actual training routine but rather undertook a protocol stipulated by researchers. This condition does not reflect the actual practices employed by physique athletes. To date, only Hackett et al. [2] specifically sought to elucidate how bodybuilders perform a routine in the OFF and PRE phases. This study surveyed 127 male bodybuilders. It is worth noting that, of 73 of the subjects who competed in non-tested amateur shows, 56 used anabolic steroids (AAS). Furthermore, training routines were not stratified between natural drug-free bodybuilders and bodybuilders who use AAS. Moreover, in the study it was also not possible to identify if the steroid users had a more efficient training strategy during the PRE phase. One of the main problems in defining a unique strategy for training routines is that male and female categories require different muscle volume and definition levels, and consequently there are very distinct training and diet strategies.

Currently, manipulating RT variables, such as intensity-load (%1 RM) and total training volume (TTV) aid in increasing the progressive overload in some manner, resulting in an anabolic stimuli to promote skeletal muscle hypertrophy [12]. These manipulations have been used for some time by bodybuilders [13]. Within these principles, athletes apply a wide range of techniques such as pyramid, negatives, supersets, and forced repetitions [13]. However, we have no reports of which techniques are most frequently utilized and how they are distributed in the training program [2]. From this perspective, it can be said that there is a glaring lack of data on the training programs and strategies that bodybuilders use in the OFF and PRE phases. Such information on bodybuilding training would be important to direct future research and assist in identifying subsequent areas of study. Thus, we aimed to perform a narrative review revisiting the training routines designed for muscle hypertrophy used by bodybuilders and provide some insight into evidence-based recommendations for bodybuilders and physique athletes.

## 2. Materials and Methods

The present manuscript reviewed the current body of evidence that examined factors related to bodybuilding, involving interventions with RT and the effects of exercise-induced muscular changes in muscular thickness, cross-sectional area (CSA), muscle strength, and body composition. The search for articles was carried out using the following databases—Pubmed/MEDLINE, Scielo, EBSCO, LILACS, SportDiscus, Web of Science, and CINAHL—which were searched from 7 January 2019 to 8 September 2020 without temporal delimitation for a broad spectrum of research. The descriptors used as search terms were: (“Body composition” OR “Muscular thickness” OR “Cross-sectional area” OR “CSA” OR “Growth” OR “Muscle growth” OR “hypertrophy” OR “Lean body mass” AND “Resistance training” OR “Resistance exercise” OR “Strength training” OR “Weight-Lifting Exercise” OR “Weight-Lifting” OR “Weight Lifting” OR “Weightlifting” OR “Strength program” AND “Bodybuilding” OR “Bodybuilder” OR “competitive body-builder” OR “physique athlete” OR “fitness competitor” OR “figure athlete”). The articles were identified and read in full, and only articles that had the words bodybuilding and training inserted in the title were selected.

Two specialists with expertise in strength and conditioning extracted the data. The information extracted from each study was: subjects, study design, program, the RT of the intervention, the results in the variables, cross-sectional area (CSA), body fat, and muscular strength.

## 3. Results

### Selected Studies Description

Fourteen studies were identified that investigated bodybuilders training routines, muscle hypertrophy, and changes in body composition. Six investigations were case studies. Only two studies had an experimental design with hypertrophy as an outcome. The descriptive observational studies presented an inadequate sample for the design. All the studies reported that the investigated athletes were champions or at least third place in their categories [14]. Only in the studies of Trabelsi et al. [15] and Gentil et al. [16] did the athletes report steroid use; all other studies involved natural bodybuilders. The observational descriptive investigations and case studies reported a broad range of preparation time that varied between 6 and 32 weeks. The studies presented in this narrative review showed similarities between the RT routines in the pre-season and off-season (Table 1).

## 4. Discussion

We reviewed the training routines used by bodybuilders. The reviewed studies demonstrate a pattern in the training organization, whereby there is a separation of training into four distinct periods: off-season, pre-contest, peak week, and post-contest. Our results demonstrated that the RT routines in these periods are composed of a range of 2–6 sets per exercise, 6–12 repetition maximum (RM), and 4–5 sets per exercise, 12–15 RM with a rest interval between sets of 90 s to three minutes.

Three studies [2,19,23] observed that athletes also perform split routines by training two muscle groups per day in the same sessions. Another pattern identified in the studies was that the competitors employed a high weekly training frequency, training five to six days per week during both the OFF and PRE.

### 4.1. Off-Season

In the OFF period, the athletes aimed to maximize their skeletal muscle hypertrophy. Alway et al. [18] investigated highly competitive (ranging from fifth to first place at National Physique Committee competitions) male and female bodybuilders with ~5 years of RT experience only during the OFF period (≥4 weeks after their most recent competition) and demonstrated a poorly detailed description of the training routine. Both genders reported that they performed 15–20 sets per exercise (chest and back) and 12–15 sets per exercise (shoulders, triceps, and biceps). The intensity-load was not mentioned, and there were no variations in the training strategies reported. Similarly, the case study by Too et al. [20] did not report the intensity load used by competitors, nor was the number of sets and repetitions performed per exercise specified. The information obtained was only about the duration and frequency of each training session, which averaged 4–5 h in 6-d·week^−1^ using a split routine program (upper body training on Monday, Wednesday, Friday; lower body training Tuesday, Thursday, Saturday; and abdominal exercises every day). The authors mentioned that the athlete performed a split training routine, but they did not make it clear if the average hours cited involved two or more training sessions per day or only one. Basically, the first 4 weeks consisted of a hypertrophy program (heavy resistance, low repetitions), followed by a 1 week transition phase to a 4-week endurance and definition program (low resistance, high repetitions), followed by a 1 week taper. Moreover, no aerobic training was incorporated in pre or post-training activities. The absence of these data in both studies makes it impossible to quantify the training volume performed by these athletes. It should be reinforced that the category, male and female, will significantly change training strategies—for example, female figure athletes utilize a higher training volume for the upper limbs as compared with bikini and wellness categories (observational evidence).

Seven reviewed studies reported further details on training routines. Manore et al. [19] and Kistler et al. [10] conducted a case study with an amateur bodybuilder who became a professional bodybuilder after 9 years of amateur career, winning the 1986 Mr. Universe, and a natural amateur bodybuilder, respectively, both with ~10 years of RT experience. Both of these monitored the training performed only in the PRE period. Manore et al. [19] reported a training frequency of four consecutive workout days and one rest day. The routines were composed of one hour of aerobic exercise (~60% VO_2_max; ~78% MHR) and three hours of RT, which was divided into morning and evening sessions. During the week, the loads were divided into light (4–6 sets per exercise; 15 reps), medium (2–6 sets per exercise; 12–20 reps), and heavy (2–6 sets per exercise; 6–10 reps). This was the only study that verified the oscillations of workloads with the intention of maximizing yield, similar to a non-linear (e.g., undulatory) periodization. Briefly, this strategy is characterized by alternating workloads (e.g., low-to-heavy or heavy-to-low), which are distributed by daily or weekly manner. Linear periodization (i.e., exponentially progression during a specific period) is another strategy that is commonly utilized; however, we did not identify this in any reviewed study. In contrast to the split-training routines reported in the aforementioned studies, a recent case study [14] reported whole-body training routines and a training frequency of 5–6 times per week for most weeks. This approach is in line with a recent meta-analysis, in which a resistance training frequency does not significantly or meaningfully impact muscle hypertrophy when the volume is equated [26].

The other reviewed studies followed with linear progressions and without mentioning any type of manipulation of RT or progression of load. Trabelsi et al. [15] observed variations in non-competitive bodybuilders training routine, in which changes that occur in body composition and markers of renal function in bodybuilders during Ramadan were verified. However, the training performed by athletes aimed to increase muscle mass. Each training session was composed of four to six exercises, with four sets of 10 RM performed per exercise and rest intervals of 2–3 min between exercises and sets. The exercises followed an execution order from multi to single-joints exercises. The division of muscle groups exercised during the week was day 1: Quadriceps, hamstrings, calves; day 2: back, triceps; day 3: Shoulder; day 4: chest, biceps. This training program was similar to the principles documented by the American College of Sports Medicine [4].

However, three studies were highlighted—one because of the size of the sample used and the other because of the use of AAS. Of the three, only two studies reported both training routines in the OFF period and in the PRE. Hackett et al. [2] evaluated 127 competitive male bodybuilders (with ~9 years of RT experience, and at least 8 bodybuilding competitions) reporting performing a 5-day split routine, averaging 60–70 min per session, and training no more than two muscle groups per session. In OFF, they performed 4–5 exercises per muscle group; 3–6 sets per exercise, 7–12 RM (higher loads) per set and 61 to 120 s recovery between sets. The PRE period utilized 4–5 exercises per muscle group, 3–4 sets per exercise, 10–15 RM (lighter loads) per set and 30–60 s recovery between sets.

Interestingly, in the study by Gentil et al. [16] in which the athletes used AAS, a training routine similar to natural bodybuilders was reported. These six amateur athletes (i.e., do not have pro card provided by federation) in the study by Gentil et al. [16] had ~10 years of experience with RT, and notably the training routines performed by these six amateur athletes used volitional failure during the execution of the exercises. Nasseri et al. [22] only assessed the hemodynamic effects in bodybuilders using EAA with a protocol proposed by the researchers, which did not accurately reflect the training reality of bodybuilding athletes. Therefore, we could not clearly observe how the training routine of the bodybuilders was used.

### 4.2. Pre-Contest

Our findings show that the primary goal during the PRE period was to reduce body fat while preserving muscle mass. During this period, RT is commonly performed with reduced loads (low-loads) and a high number of repetitions with short interset rest intervals (30 to 60 s). This training strategy was done because some athletes believed that performing more repetitions with lower load and shorter intervals would be more efficient for preserving skeletal muscle mass. Additionally, in this period, the dietary restriction may induce the use of lower loads. Another possible explanation for high volume low-load training during this phase is the believe of body fat reduction with this strategy. Observational evidence may also indicate that some athletes choose to maintain high loads, decreasing the training volume, while using some sets near to failure, and with intraset rest intervals due to fatigue and tiredness induced by severe caloric restriction. The weeks before competing also involve protocols to induce dehydration and a very low carbohydrate intake, which may also contribute to these training strategies [27].

In PRE, subjects train under severe caloric deficits associated with a greater volume of aerobic exercise, which can potentially compromise fat-free mass. This caloric deficit also induces a decrease in training intensity. This leads the athletes to imagine that decreased load is the better strategy to preserve the gains obtained in OFF. This training configuration increases TUT and metabolic stress, which can stimulate increases in acute protein synthesis and contribute to complex physiological mechanisms responsible for muscle hypertrophy [28]. Moreover, the metabolic stress induced by RT involves an increase in intracellular hydration and the raising of the water content of the muscle cells, which also has been suggested as an important stimulus for muscle growth in the condition of higher metabolite accumulation [29]. In this sense, a feasible strategy for maximize hypertrophy gains during the pre-contest period would be low-load training associated with blood flow restriction. The blood flow restriction associated with exercise-induced metabolic stress may expose muscle fibers (specially type I fibers) to a new recruitment pool that could not be reached during RT with heavy-loads, hence eliciting a distinct anabolic stimuli to induce hypertrophy gains in well-trained athletes [30].

Nevertheless, these bi-seasonal training programming differences were consistently seen across studies. Additionally, in the PRE, all studies reported that athletes included aerobic training with RT to maximize the reduction in body fat. Although the initial data on the training routines presented similarities, the individual examination of the studies revealed differences in the protocols, and the monitoring period investigated. Of the 13 studies reviewed, four [17,18,19,20] were from the past century and did not have a detailed methodological description as the investigations from the 2000s. This made it difficult to report detailed training routine information. Additionally, Escalante and Barakat [31] reviewed fasted versus nonfasted aerobic exercise on body composition for physique athletes, and stated the difficult to discuss the real effects of fasted versus nonfasted aerobic exercise due to methodological aspects (i.e., controlled studies). In this sense, the hypothesis of low glycogen levels after an overnight fast allowing greater stored fat mobilization to be used for fuel remains inconclusive.

### 4.3. Peak Week

While a majority of the studies did not report a clear difference in the training routine performed between PRE and the week prior to the contest, also known as peak weak, the athletes has related particular short-term strategies to achieve the leanest possible appearance, as water and sodium manipulation, as well as carbohydrate loading regimen [3,32]. Although nutritional manipulations are not the scope of this review, the training regimen may be altered to accommodate peaking strategies.

In these sense, only a case study [14] with a 10-year experienced lifter related in detail the strategies during peak week. In this, the athlete performed a carbohydrate loading protocol, which consisted of a 3-day carb depletion phase (<50 g per day). followed by a 2-day carb loading phase (>450 g per day). The number of exercises was gradually reduced over the peak week. On depletion days, walking was performed for 40–90 min, reducing to 15 min or no exercise on loading days. These reductions in volume and intensity may be necessary for minimizing the possibility of inflammation and edema as a consequence of muscle-damaging exercise, which may impair glycogen synthesis [33] and accumulate subcutaneous water into the interstitial tissues [34], potentially worsening muscle definition. The study of Mitchell et al. [25] also shows a reduction in training volume, which was prominently for the lower limbs.

Thus, during the peak week, the training may be altered to accommodate peaking strategies, such as carbohydrate loading, due to the fact that this strategy in particular may increase muscle size [3,14] while pulling subcutaneous water into the muscle [35], thereby achieving a greater muscle size and the defined appearance coveted by bodybuilders.

### 4.4. Post-Competition

Post competition is a phase in which faster weight gain occurs; freedom in terms of diet and a reduction in training volume and intensity help restore a competitor to a healthy status both psychologically and physiologically [32]. While it has been reported that the post competition period comprises the 4 weeks after the main event [32], it can extend to up to 5–6 months [24,36]. Four case studies following drug-free athletes in this period were found [14,20,24,36]. All the studies documented outcomes in body composition, muscle strength, and exercise capability, while only Schoenfeld et al. [14] included site-specific measures of muscle growth.

In the study of Too et al. [20] the post-competition activity consisted of 1 week of rest (complete inactivity), followed by 4 weeks of very light resistance training (l-h in duration, 3-d·week^−1^). No aerobic exercise was incorporated in either pre- or post-training activities, resulting in the rapid return of both CK and lactate dehydrogenase to normal values within l week post competition.

In contrast, a professional bodybuilder studied by Rossow et al. [36] seems to have taken longer to restore psychological and physiological changes after the competition. The subject performed 4 days of resistance training (total 5 h/week) and 1 day of high-intensity interval training (total 20 min/week). The resistance-training frequency for each major muscle group remained at twice per week during this period. Despite having decreased aerobic exercise and the resistance exercise remaining relatively constant, squat and deadlift strength (absolute 1-repetition maximum) and critical power (cycle ergometer) recovered by month 4, while bench-press strength and critical power remained below baseline at month 6. This lack of exercise capability recovery coincided with depressed vigor and fatigue may reflect psychological alterations.

In the study of Pardue et al. [24] who assessed an amateur drug-free male bodybuilder with 8 years of RT experience and one year of competitive bodybuilding experience, there was no detailed description of training routines after competition. However, the authors reported that a drop observed in the peak power and average power output (Wingate test) had not fully returned at 5 months after competition.

**Figure 1:**
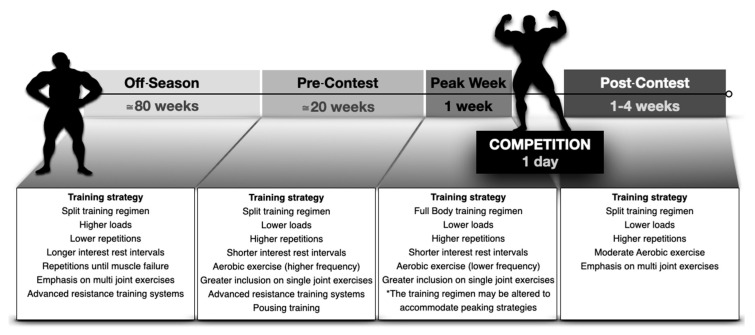
Schematic model of the training routines adopted by bodybuilders with the main characteristics of each preparation phase.

Ultimately, Schoenfeld et al. [14] reported an athlete competing in four successive events within a few weeks and 4 months after the last competition. The authors reported that muscle thickness (analyzed by mode-B ultrasound) quickly recovered during the first month post competition with marginal alterations until the fourth post-competition month. Moreover, maximum isometric strength of the knee extensor (dynamometry) decreased by 9% and explosive strength (vertical jump height) increased by 18% four months after the last competition. With the exception of the study of Too et al. [20], the findings suggest that the athletes showed symptoms of overtraining to a greater or lesser extent, probably reflecting the restrictive practices conducted prior to the competition. In summary, Figure 1 shows a timeline design of bodybuilders’ training routines.

### 4.5. Future Perspectives

Although the mention of bodybuilders is recurrent in the context of studies that investigate the effectiveness of advanced strength training techniques, there are no randomized controlled studies in the literature with samples composed even of amateur athletes. Two recent reviews on resistance training systems have concluded that, to date, there is insufficient evidence to determine whether RT systems can maximize hypertrophy and muscle strength when compared to traditional resistance training [5], despite the fact that RT systems may be feasible strategies to avoid monotony, reduce training duration, and avoid plateaus in muscular adaptation [6]. However, in both reviews there is no mention of any study conducted with bodybuilders.

In fact, studies carried out with untrained individuals or even “recreational trained” ones, although relevant to test the specific hypothesis, cannot serve to justify inferences about the usefulness of alternative training strategies adopted by bodybuilders.

Furthermore, the difficulty in developing guidelines (i.e., intensity, volume, training division, the characterization of methods, periodization models) for training prescription aimed at bodybuilders, far beyond what is suggested in this paper, is worth noting, given the diversity of strategies adopted by athletes and coaches, for which the methodological variables of strength training are manipulated without apparent predictability or systematization. However, it is noteworthy that training based on adaptations of recreationally trained or untrained individuals may not be an exact fit for “bodybuilders” yet, but the physiological adaptations and increases in the markers of skeletal muscle hypertrophy can certainly be made to help inform practice.

It should be noted that the goals pursued by bodybuilders (muscular mass, symmetry, and definition) focus on the morphological adaptations of the skeletal muscle, and, in this scenario, an increase in muscle strength is not even a priority. Therefore, classic resistance training periodization is not always strictly observed, and specific biomechanical strategies are employed, albeit empirically, to obtain hypertrophic responses from specific muscle groups. In fact, the indiscriminate gain in muscle volume does not seem interesting for this population, given that intra and intermuscular hypertrophy responds differently to different exercise modalities [37,38,39,40].

Choosing the adequate exercise type might be crucial point for a bodybuilder’s success or failure. In the search for symmetry, for example, especially in the PRE period, the need to “correct” the flawed points can compel the athlete to prioritize certain muscles over others (i.e., gluteus maximus over the quadriceps; rectus femoris over the vastus; distal portion of the vastus lateralis to the detriment of the proximal portion).

In this scenario, case studies and observational studies are particularly relevant in the literature, given the difficulty of recruiting samples composed by bodybuilders involved in competitions and allocating them to training conditions that, at first, escape routine or conflict with strategies typically adopted individually by these individuals or by their coaches.

Therefore, future studies can investigate the effectiveness of strategies narrated or practiced by the athletes themselves or by their coaches, including the assessment of any heterogeneous hypertrophic responses with the aid of images (i.e., magnetic resonance, ultrasound). Additionally, survey-based studies with a large sample size constituting male and female bodybuilders may be a feasible strategy to understand some details of bodybuilders’ training routines, such as the use of specialized techniques and RT systems. The results may support the development of new hypotheses, which can be tested on trained individuals, but without competitive pretensions.

## 5. Conclusions

Most of the reviewed studies were case reports or contained small numbers of participants; all the athletes investigated had significant titles in high-level competitions. With this, we detailed the various training routines performed by the champions (natural bodybuilders) of their categories. Although there was a high degree of heterogeneity across the training protocols, recurrent themes among bodybuilding competitors in OFF were higher loads/lower repetitions and longer interest rest intervals than PRE. In addition, bodybuilders commonly performed aerobic exercise in PRE. Competitors unanimously employed split-body rather than full-body training regimens. It is likely that athletes in lower-level competitions perform training routines similar to those reported in our review. On the other hand, the athletes who use AAS have training routines that largely remain uninvestigated, indicating that further studies into this population are necessary. Finally, from our own daily experience with bodybuilders of different categories we can also report that athletes are likely to use many training methods, some of them with very short rest intervals between sets (e.g., <30 s), and may incorporate different strategies for each muscle group. For example, the weakest muscle (at competitive parameters) would be trained more frequently.

## Figures and Tables

**Table 1:** Methodological characteristics of the studies.

Authors (year)	Participants	Design	Training Program
Sandoval et al. (1989) [17]	n = 5 (male), 25 ± 3.3 years and 3.6 (range: 1–10) years of experience in bodybuilding competitions; weight (kg): 82.2 ± 9.7 n = 6 (female), 27.8 ± 4.1 years and 1.7 (range: 0.75–2.5) years of experience in bodybuilding competitions; weight (kg): 52.2 ± 3.1	Athletes responded to questionaries’ concerning their experiences in sport and training program 24–48 h prior to contest.	Male: 5.5 days/week; 9.5 h/week; range 10–13 sets/muscular group with exception abdomen (6.7 sets) and 9–10 maximum repetitions/set with exception abdomen (26.2 repetitions/set); Female: 5.8 days/week; 13.8 h/week; range 13–23 sets/muscular group and 12–13 maximum repetitions/set; 60% of the men and 100% of the woman performed aerobic exercise with duration of 1.5–3 h/week and 1.5–3.6 h/week for men and women, respectively.
Alway et al. (1992) [18]	n = 5 (male) n = 5 (female) n = 2 (control) Time of training: −5.8 ± 0.5 (male) −5.4 ± 0.7 (female) Did not use steroids during the investigation period.	Duration 12–24 weeks (program training). Frequency of training 5–6 days/weeks Not intervention in study, only observation. Request information by questionnaire. Athletes were examined ≥4 weeks after their most recent competition.	15–20 sets per exercise (chest and back). 12–15 sets per exercise (shoulder, triceps and biceps).
Manore et al. (1993) [19]	n = 1 (male)—31 years Stature: 171.5 cm Weight: 95 kg (off-season) Weight: 88.5–90 kg (pre-contest) Did not use steroids during the investigation period.	Case study: only observation. Information was verbally requested to follow the training program. Exercise program training consisted of 2 h of aerobic activity and 3 h of strength training/day. 4 consecutive workout days and 1 rest day. The athletes were studied for 8 weeks before his first competition of the season.	1 h/day aerobic (cycling ergometric) 1 h/day aerobic (cycling bike) Both in ~60% VO2max (~78% MHR). Strength training was divided in light, medium and heavy weights. The split session in morning and evening. Light = 4–6 sets per exercise (15 reps.) Medium = 2–6 sets per exercise (12–20 reps.) Heavy = 2–6 sets per exercise (6–10 reps.)
Too et al. (1998) [20]	n = 1 (male)—34 years (Asian) Weight: 76.3 kg (off-season) Weight: 71.6 kg (pre-contest) Did not use steroids during the investigation period.	His bodybuilding regimen during a 15-year period consisted of a whole body workout with heavy strength training and low repetitions. The researchers only observed. During initial monitoring (10 week) of the study the athlete decided to training with a periodized bodybuilding program that was advertised in a popular bodybuilding magazine.	10 week the training program was used, that was consisted of daily strength training sessions 4–5 h in duration 6 days/week used a split routine program (upper body on Monday, Wednesday, and Friday; lower body on Tuesday, Thursday, and Saturday. Abdominal every day). The first 4 weeks consisted of hypertrophy (heavy resistance and lower repetition), 1-week transition phase to 4-week endurance and definition phase (lower resistance and high repetition)
Trabelsi et al. (2012) [15]	n =16 (male) n = 9 (fasters) 24 ± 3 years 79.9 ± 5.1 kg 175 ± 5 cm n = 7 (non-fasters) 26 ± 3 years 81.5 ± 5.4 kg 177 ± 3 cm Both groups without mention of steroid use.	Verified the changes that occur in body composition and markers of renal function in bodybuilders during Ramadan without intervention by the researchers. The training program consisted of workouts using exercises with free weights and machines. The primary goal of the program was to increase muscle mass (hypertrophy). They were followed for two weeks, which is the Ramadan period.	Each training session was composed of four to six exercises. Each exercise was performed in four sets with a load of 10 RM and intervals of 2–3 min between exercises and sets. The exercises were conducted first with the major muscle groups and then with the smaller muscle groups. The training intensity was increased progressively. Day 1: Quadriceps, hamstring, calves; Day 2: Back, triceps; Day 3: Shoulder; Day 4: chest, biceps. The program closely followed the principles documented by the American College of Sports Medicine.
Hackett et al. (2013) [2]	n = 127 (male) 28.7 ± 6.3 years 177.5 ±11.8 cm 96.6 ± 7.7 kg Not all participants were tested for drugs in competitions.	Not intervention in study, only observation. The study proposed to describe training practices by competitive bodybuilders and to determine whether training practices comply with the current recommendations for muscular hypertrophy. A web-based application (Survey Monkey) was used to assess the training practices used by the bodybuilders. The URL address of the survey was made available to potential subjects through links or postings placed on various bodybuilding websites.	Training session durations ranged between 40 and 90 min. The elite bodybuilders reported performing a 5-day split routine, averaging 60–70 min per session, and training no more than 2 muscle groups per session. Off-Season: 4–5 exercises per muscle group, 3–6 sets per exercise, 7–12 RM (higher loads) per set and 61 to 120 s recovery between sets. Pre-Contest: 4–5 exercises per muscle group, 3–4 sets per exercise, 10–15 RM (lighter loads) per set and 30–60 s recovery between sets.
Kistler et al. (2014) [10]	n = 1 (male) 26 years 10 years resistance training experience. A prior not use steroids	Case Study: only observation. Information was verbally requested to follow the training program. The subject tracked his diet and exercise training throughout the study. Resistance training was performed 5 days of the week, approximately 1 to 1.5 h per day, throughout contest preparation. Each muscle group was trained twice weekly.	1 day primarily in the 3 to 8 repetition range and the other primarily in the 8 to 15 repetition range. This quantity of resistance training was maintained throughout the preparation. During the 26-week preparation, the athlete and attempted to maintain resistance-training load. At the start of the contest preparation, two 40 min sessions of HIIT were performed per week. This HIIT generally consisted of a 30 s all-out sprint, followed by 4:30 of active jogging recovery. At the end of contest preparation, the subject performed four 60 min sessions of HIIT and two 30 min sessions of low-intensity steady-state aerobic exercise per week.
Robinson et al., 2015 [21]	21-year-old male amateur bodybuilder 2 years resistance training experience 14 weeks prior to his first bodybuilding competition in the Men’s Physique category.	Case Study: 14-week intervention	Four RT sessions during each week of the intervention; targeting each major muscle group on two occasions per week. Each RT consisted of 6–8 exercises performed for 8–10 repetitions and 4–5 sets. A combination of high intensity interval training (HIIT) and low-intensity steady-state (LISS) exercise were performed in the overnight fasted state.
Gentil et al. (2017) [16]	n = 4 (male) n = 2 (female) The participants make use of anabolic.	This is an observational study. All the data were provided by the participants and their coaches after the competition. Bodybuilders and their coaches were requested to describe in detail all their practices (training, diet, nutritional supplements and pharmacological agents). When any doubt arose, competitors/coaches were directly contacted to give further details.	Each muscle group once a week with multiple sets of multi- and single-joint exercises performed to volitional fatigue. In the bulking phase, the male and female athletes performed sets of 8–12 repetitions with 2–3 min of rest between sets. In the cutting phase, the number of repetitions increased to 12–15, and the rest intervals dropped to 45–60 s. Increased the time spent in fasted cardio during cutting (45–60 min of cardio bicycle/treadmill at moderate intensity).
Nasseri et al. (2015) [22]	n = 8 (steroids users) 27.4 ± 2.9 years 176 ± 0.05 cm 80.5 ± 10.3 kg n = 8 (steroids non users) 27.8 ± 2.2 years 181 ± 0.06 cm 80.8 ± 9.3 kg	The study, as part of a clinical trial, aimed to explore how one session of resistance exercise affects the hemodynamic characteristics (i.e., HR and BP) and the levels of the muscle damage markers (i.e., CK and LDH enzymes) in professional bodybuilders who were AAS users.	Circuit resistance training session composed of 7 stations including leg press, bench press, leg extension, lat pulldown, leg curl, shoulder press and biceps curl exercises. The exercise session involved 3 sets of 8–9 repetitions at 80–85% of 1 RM. Rest intervals between sets and the stations were considered 60 and 90 s, respectively. A warm-up of 15 min was performed before the exercise, begin that 5-min jogging on treadmill (speed 7 km/h, 1.5% inclination), and a specific warm-up including two sets of the exercises, same to those performed in the training session, using 35% (15 reps) and 45% (12 reps) from 1 RM.
Syed-Abdul et al. (2019) [23]	Case study n = 2 (male steroids non users) “P1”: 21 years, 82.6 kg 22 years, “P2”: 89.6 kg	Case Study: 8 weeks prior to competition. Information was self-reported. Athletes was evaluated (food intake, body composition) pre and post 5 weeks with self-implemented carb cycle restrictive diet and exercise program.	P1: performed high-intensity [60–90 min, 75–90% of one repetition max (1 RM)] resistance training and P2: moderate-intensity (30–45 min, 60–75%1 RM) resistance training 2 h of aerobic activity and 3 h of strength training/day. Four consecutive workout days and 1 rest day.
Pardue et al. (2017) [24]	A case study with 21-year-old, amateur, drug-free male bodybuilder with eight years of weight training experience and one year of competitive bodybuilding experience.	Blood samples were taken approximately every three months for hormone analysis; body composition, anaerobic power, resting metabolic rate and sleep quality were assessed monthly during the pre-contest phase (8 months), followed by recovery (5 months).	Resistance training was performed 5–6 days per week. A mixture of multi- and single-joint exercises was completed with a variety of repetition ranges (4–25 repetitions) and intensities. Major muscle groups were trained at least twice per week, with ≥48 h of rest between training sessions of the same muscle group. Aerobic activity was adjusted each week based on weight loss progress. Aerobic activity consisted of moderate intensity steady state (MISS) cardiovascular exercise at 65–75% of maximal heart rate, along with sessions of high intensity interval training (HIIT) with 10–15 s bouts of maximal effort separated by 45–50 s active recovery intervals.
Mitchell et al. (2018) [25]	Male bodybuilders steroids non users (n = 9) 29.0 ± 9.5 years, 177.9 ± 2.5 cm, 83.7 ± 8.9 kg, 6.0 ± 6.6 years bodybuilding participation).	Body composition, resting metabolic rate (RMR), serum hormones, and 7-day weighed food, resistance training volume (repetitions × weight × sets) and training diaries of natural male bodybuilders (n = 9) were assessed 16 (PRE16), 8 (PRE8), and 1 (PRE1) week(s) before, and 4 (POST4) weeks after a bodybuilding competition.	Upper body volume (kg × week^−1^): 39,958 ± 17,232 (PRE 16); 42,368 ± 19,647 (PRE 8); 32,753 ± 14,385 (PRE 1); 37,432 ± 15,384 (POST 4); Lower body (kg × week^−1^) 42,503 ± 24,234 (PRE 16); 51,247 ± 37,997 (PRE 8); 33800 ± 33,697 (PRE 1); 41,735 ± 34,225 (POST 4);
Schoenfeld et al. (2020) [14]	n = 1 (male) 25 years 10 years resistance training experience. A prior not use steroids (drug-tested)	Prospective case study in a high-level amateur male bodybuilder throughout preparation for 4 competitions and during the ensuing post-contest recovery period, totaling 1 year. They analyzed the muscle thickness in 4 sites, body composition, hemodynamic characteristics (BP and HR), resting metabolic rate, vertical jump height, isometric lower body strength testing, and a 3-factor eating questionnaire. Blood collections for hormones and enzymes analysis was obtained separately from an outside laboratory at 4 time points.	Three to 7 days/per week (5–6 times per week for the majority); generally, whole-body training routines, 10–14 exercises; 1–10 sets/exercise (3–4 in the majority), 3–30 repetitions (6–15 in the majority) with higher number of repetitions generally performed only for single-joint exercises, whereas a lower number of repetitions was generally performed for multi-joint exercises; carried out to repetitions in reserve (RIR) 1, RIR 2, or RIR 3 and RIR 4 during deload sessions (25/225); 30 min of daily walks and during peak weeks, the walking duration was altered to accommodate carbloading strategies.

Abbreviations: VO2max: maximum oxygen consumption; 1 RM: one repetition maximum; MHR: maximal heart frequency; HIIT: high intensity interval training; Reps: repetition; HR: heart frequency; BP: blood pressure; CK: creatine kinase; LDH: lactate dehydrogenase; AAS: anabolic androgenic steroids; RMR: resting metabolic rate; RIR: repetitions in reserve; HIIT: high-intensity interval training; MISS: moderate-intensity steady-state; LISS: low-intensity steady-state.
